# Physical Mapping of a Novel Locus Conferring Leaf Rust Resistance on the Long Arm of *Agropyron cristatum* Chromosome 2P

**DOI:** 10.3389/fpls.2018.00817

**Published:** 2018-06-18

**Authors:** Bo Jiang, Taiguo Liu, Huanhuan Li, Haiming Han, Lihui Li, Jinpeng Zhang, Xinming Yang, Shenghui Zhou, Xiuquan Li, Weihua Liu

**Affiliations:** ^1^National Key Facility for Crop Gene Resources and Genetic Improvement, Institute of Crop Sciences, Chinese Academy of Agricultural Sciences, Beijing, China; ^2^State Key Laboratory for Biology of Plant Diseases and Insect Pests, Institute of Plant Protection, Chinese Academy of Agricultural Sciences, Beijing, China; ^3^College of Life Sciences, Henan Agricultural University, Zhengzhou, China

**Keywords:** *T. aestivum*–*A. cristatum* 2P chromosome translocation lines, leaf rust resistance, physical mapping, molecular marker, novel disease-resistant germplasm

## Abstract

Wheat leaf rust is one of the most common wheat diseases worldwide and can cause up to 40% wheat yield loss. To combat the growth and spread of leaf rust disease, continual exploration and identification of new and effective resistance genes are needed. Here, we report for the first time a locus conferring leaf rust resistance located on the long arm of *Agropyron cristatum* chromosome 2P in *Triticum aestivum*–*A. cristatum* 2P translocation lines. This study used 50 leaf rust races, including two Chinese major dominant leaf rust races, named by THT and PHT, and other 48 different leaf rust races collected from 11 provinces, 1autonomous region and 1 municipality of China to test the resistance to *T. aestivum–A. cristatum* 2P chromosome translocation lines and their backcross populations, the results indicated that the novel leaf rust resistance locus was immune or nearly immune to all tested leaf rust races. Four long arm translocation lines with different breakpoints of *A. cristatum* chromosome 2PL and their backcross populations were tested with leaf rust race THT at the seedling and adult stages and genotyped with 2P-specific STS markers. The results showed that the novel leaf rust resistance locus of the *T. aestivum*–*A. cristatum* 2P translocation lines was located in the chromosomal bin FL 0.66–0.86 of 2PL. Therefore, *T. aestivum–A. cristatum* 2P chromosome translocation lines conferring leaf rust resistance locus could provide a novel disease-resistance resource for future wheat breeding programs.

## Introduction

Common wheat (*Triticum aestivum* L., 2*n* = 6*x* = 42, genomes AABBDD) is one of the most important widely planted food crops worldwide (Su et al., 2013). Wheat production plays an important role in the development of the national economy (Costanzo and Barberi, 2014). Wheat leaf rust caused by *Puccinia triticina* Erikss. is one of the most common diseases of wheat throughout the world (Bolton et al., 2008; Kolmer, 2013), and serious cases can cause up to 40% wheat yield loss (Knott, 2012). Recent changes in climate conditions caused by global warming are more suitable for the spread of wheat leaf rust, increasing concern.

Wild relatives of common wheat contain a large number of desirable genes that can be exploited for wheat improvement (Li et al., 2017). The discovery and utilization of the desirable genes from wild relatives is significant for the genetic improvement of wheat resistance. At present, 76 wheat leaf rust resistance genes have been named and identified in common wheat and its relatives (McIntosh et al., 2016; Bansal et al., 2017). Approximately half of the leaf rust resistance genes are derived from wild relatives of common wheat, including *Aegilops* L., Secale cereale L., and *Elytrigia* Desv.. *Aegilops* L. is the donor of 15 leaf rust resistance genes described in the Catalog of Wheat Gene Symbols, including *Lr9*, *Lr21*, *Lr22a*, *Lr28*, *Lr29*, *Lr32*, *Lr35*, *Lr36*, *Lr37*, *Lr39*, *Lr42*, *Lr47*, *Lr51*, *Lr66,* and *Lr76* (Marais et al., 2010; Martynov et al., 2015; Bansal et al., 2017). *Lr25* (Singh et al., 2012), *Lr26* (Singh et al., 2006), and *Lr45* (Friebe et al., 1996; Naik et al., 2015) were transferred from *S. cereal* L., and *Lr19* (Sarma and Knott, 1966), *Lr24* (Hart et al., 1976) and *Lr38* were transferred from *Elytrigia* Desv. (Friebe et al., 1992, 1993; Mebrate et al., 2007). These genes have played very important roles in wheat resistance breeding, but many of them have been defeated by new virulent races. Therefore, new and effective resistance genes constantly need to be explored and identified by breeders and researchers to combat the growth and spread of leaf rust disease.

*Agropyron* Gaertn., one of the important wild relatives of common wheat, is a useful genetic resource for wheat genetic improvement (Dewey, 1984; Dong et al., 1992; Lu et al., 2015). The exploitation of desirable genes from the P genome of *Agropyron cristatum* has mainly been reported by our laboratory. For example, *A. cristatum* chromosome 2P shows high resistance to powdery mildew caused by *Blumeria graminis* f. sp. *tritici* (*Bgt*) (Li Q. et al., 2016, Li et al., 2017). *A. cristatum* chromosome 7P confers higher tolerance to drought compared with Fukuho (Lu et al., 2016). *A. cristatum* chromosome 6P contains genes conferring high numbers of kernels per spike genes (Wu et al., 2006; Han et al., 2014). Besides, it was also found that *A. cristatum* chromosome 6P carries leaf rust resistance gene(s). The leaf rust resistance gene(s) was mapped to the bin 6PS-0.81-1.00 using the BC_1_F_2_ population of 6PS/6PL telosomics at the adult plant stage (Song et al., 2016). Moreover, *T. aestivum*–*A. cristatum* 2P addition line was induced by ^60^Co-γ ray and *Aegilops cylindrical* gametocidal chromosome 2C, leading to the creation of *T. aestivum*–*A. cristatum* 2P translocation lines and deletion lines with different chromosomal fragments and breakpoint positions (Li H. et al., 2016, Li et al., 2017). Then a physical map of *A. cristatum* chromosome 2P was constructed with specific STS (sequence-tagged site) markers (Li et al., 2017). In addition, we previously showed that the *T. aestivum*–*A. cristatum* 2P addition lines are more resistant to leaf rust than the *T. aestivum*–*A. cristatum* 6P addition lines. We determined that *T. aestivum*–*A. cristatum* 2P addition line II-9-3 is not only highly resistant to powdery mildew but also shows good resistance to leaf rust.

In this study, *T. aestivum*–*A. cristatum* 2P disomic addition lines II-9-3 and translocation lines were inoculated with different races at the seedling stage to identify the resistance spectrum and availability of II-9-3 against leaf rust. Then, the BC_2_F_2_ or BC_3_F_2_ populations of *T. aestivum*–*A. cristatum* 2P translocation lines with different chromosomal segments were used to evaluate the resistance and genotype with molecular markers to map the novel locus to a specific region of chromosome 2P. These data provide a scientific basis for effectively using this novel leaf rust resistance locus in wheat improvement and basic research.

## Materials and Methods

### Plant Materials

The plant materials in this study included *T. aestivum* cv. Fukuhokomugi (Abbreviation: Fukuho, 2*n* = 6*x* = 42, genomes AABBDD); *T. aestivum*–*A. cristatum* 2P disomic addition line II-9-3 (2*n* = 44); 5 *T. aestivum*–*A. cristatum* 2P translocation lines 2PT-3 (4DS.2PL), 2PT-5 [3BS.L-2PL(0.6-1)], 2PT-6 [6AS.L-2PL(0.37-0.66)], 2PT-8 [4AS.L-2PL(0.86-1)], and 2PT-10 (2PS.1AL); and 5 backcross populations derived from the crosses between the 5 translocation lines (2PT-3, 2PT-5, 2PT-6, 2PT-8, and 2PT-10) and Fukuho. The sizes of the 2PT-3 and 2PT-10 BC_1_F_2_ backcross populations were 131 and 211, respectively. At the seedling stage, each of the 2PT-3, 2PT-5, and 2PT-6 BC_3_F_2_ or BC_2_F_2_ backcross populations had a size of 200, and the size of the 2PT-8 BC_2_F_2_ backcross populations was 196. At the adult plant stage, the size of the 2PT-5, 2PT-6 and 2PT-8 BC_2_F_2_ backcross populations were 153, 126, and 106 respectively. The size of the 2PT-3 BC_2_F_2_ backcross populations was 159 at the adult plant stage test. The numbers of positive and negative plants of each population were showed in **Tables 1**, **2**. These materials were provided by the Wheat Resources Laboratory, Institute of Crop Sciences, Chinese Academy of Agricultural Sciences. The susceptible control *T. aestivum* cv. ‘Zhengzhou5389’ was kindly provided by the State Key Laboratory for Biology of Plant Diseases and Insect Pests, Institute of Plant Protection, Chinese Academy of Agricultural Sciences.

**Table 1:** Response to leaf rust race THT and molecular marker analysis of the 2PL translocation line populations in the field.

Parents and populations	Type of translocation line	Number of detected plants	Number of plants of 2P-specific markers	Number of resistance and susceptible plants
II-9-3		30	+ (30)	R(30) S(0)
Fukuho		30	-(30)	R(0) S(30)
2PT-10/Fukuho BC_1_F_2_	2PS.1AL	211	+ (102)	R(0) S(102)
			-(109)	R(0) S(109)
2PT-3/Fukuho BC_1_F_2_	4DS.2PL	131	+(97)	R(97) S(0)
			-(34)	R(0) S(34)

*R indicates resistance; S indicates susceptible; ‘+’ indicates the progenies of the translocation lines and their parents containing A. cristatum 2P chromatin; ‘-’ indicates the progenies of the translocation lines and their parents that do not contain A. cristatum 2P chromatin*.

**Table 2:** Response to leaf rust race THT and molecular marker analysis of the 2PL translocation line populations at the seedling and adult plant stages.

Materials and populations	Seedling stage			Adult plant stage		
	Number of detected plants	Number of plants of 2P-specific markers	Number of resistance and susceptible plants	Number of detected plants	Number of plants of 2P-specific markers	Number of resistance and susceptible plants
Zhengzhou5389	30	-(30)	R(0) S(30)	30	**-**(30)	R(0) S(30)
II-9-3	30	+(30)	R(30) S(0)	30	+(30)	R(30) S(0)
Fukuho	30	-(30)	R(0) S(30)	30	-(30)	R(0) S(30)
2PT-3/Fukuho BC_3_F_2_	200	+(142)	R(142) S(0)	159	+(106)	R(106) S(0)
		-(58)	R(0) S(58)		-(53)	R(0) S(53)
2PT-5/Fukuho BC_2_F_2_	200	+(145)	R(145) S(0)	153	+(103)	R(103) S(0)
		-(55)	R(0) S(55)		-(50)	R(0) S(50)
2PT-6/Fukuho BC_2_F_2_	200	+(108)	R(0) S(108)	126	+(64)	R(0) S(64)
		-(92)	R(0) S(92)		-(62)	R(0) S(62)
2PT-8/Fukuho BC_2_F_2_	196	+(110)	R(0) S(110)	109	+(51)	R(0) S(51)
		-(86)	R(0) S(86)		-(58)	R(0) S(58)

*R indicates resistance; S indicates susceptible; ‘+’ indicates the progenies of the translocation lines and their parents containing A. cristatum 2P chromatin; ‘-’ indicates the progenies of the translocation lines and their parents that do not contain A. cristatum 2P chromatin. The numbers in the table indicated the number of the progenies of the translocation lines corresponding to resistance or susceptible to leaf rust.*

### Collection of the Leaf Rust Isolates

**FIGURE 1:**
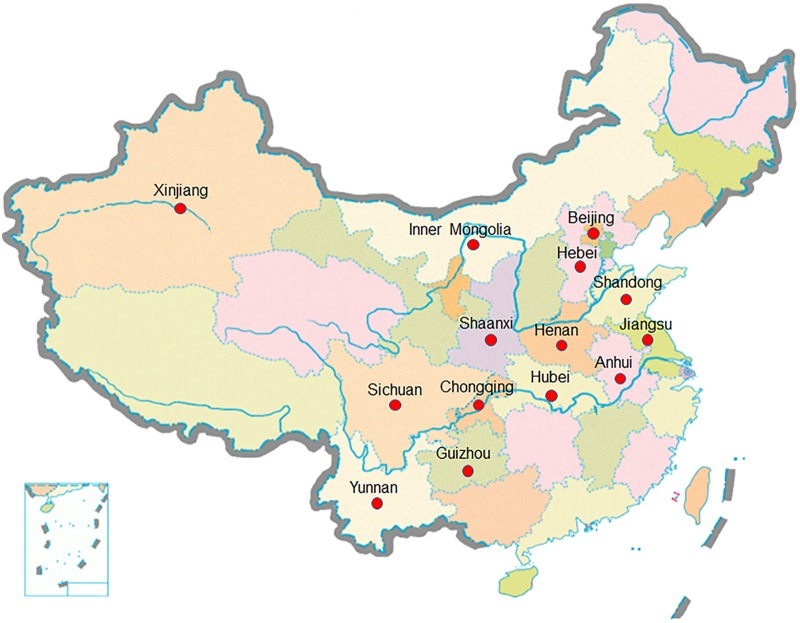
Map of China. The provinces, autonomous regions or municipalities from which the collections of *Puccinia triticina* were obtained are marked out.

This study used 50 leaf rust races, including two Chinese major dominant leaf rust races, named by THT and PHT, and other 48 different leaf rust races collected from different locations in Hebei, Henan, Shandong, Inner Mongolia, Jiangsu, Anhui, Hubei, Shaanxi, Xinjiang, Yunnan, Guizhou, Sichuan, and Chongqing of China in 2016 (**Figure 1**). The 50 races tested were identified with a total of 102 samples collected from the mentioned 11 provinces, 1 autonomous region and 1 municipality, and the detailed information was listed in **Table 3**. The 50 races were denominated using the Prt code system (Long and Kolmer, 1989) by Liu and Chen (2012). The leaf rust races used in this study were kindly provided and tested by the State Key Laboratory for Biology of Plant Diseases and Insect Pests, Institute of Plant Protection, Chinese Academy of Agricultural Sciences, China.

**Table 3:** Infection types of the *T. aestivum*–*A. cristatum* 2P addition line and the 2PL translocation line for different leaf rust races at the seedling stage.

Leaf rust collect locations		Races	Zhengzhou5389	Fukuho	II-9-3	2PT-5
Provinces	City/County/Village					
Hebei	Xinle^a^	PHK	3	3	0	0


		PCH	3^+^	3^+^	0	0


	Wuji^b^	PHT	3	3	0	0


	Ningjin^b^	SHK	3^+^	3^+^	0	0


		SHT	3	3	0	0


	Shenze^b^	PHK	3^+^	3^-^	0	0


		PCH	3^+^	3^+^	0	0


	Hengshui^a^	CGJ	3	3	0	0


	Jindou^a^	PCH	3	3	0	0


Henan	Zhang xiaoying^d^	MCG	3	3	0	0


		PHK	3^+^	3^+^	0	0


	Baitugang^d^	PCG	3	3	0	0


		PHK	3	3	0	0


	Sizhai^d^	THK	3	3	0	0


	Liujiadi^d^	PHK	3^+^	3^+^	0	0


	Zhoukou^a^	FCH	3^+^	3^+^	0	0


		THK	3	3	0	0


	Guoyang^b^	PCT	3	3	0	0


		PCH	3	3^+^	0	0


	Nanyang^a^	PCG	3	3	0	0


		PHK	3^+^	3^+^	0	0


	Tanghe^b^	PHJ	3^+^	3^+^	0	0


	Xinyang^a^	PCG	3	3	0	0


	Luoyang^a^	PHK	3	3	0	0


	Neihuang^b^	MHJ	3	3^+^	0	0


Shandong	Linyi^a^	PHT	3^+^	3^+^	0	0


		PHJ	3^+^	3^+^	0	0


		NHJ	3^+^	3^+^	0	0


		PHS	3^+^	3^+^	0	0


		THT	3^+^	3^+^	0	0


Inner Mongolia (autonomous regions)	Hohhot^a^	FHL	3^+^	3^+^	0	0


		THJ	3	3	0	0


		PHT	3^+^	3^+^	0	0


		PHS	3^+^	3^+^	0	0


		MHK	3^+^	3^+^	0	0


		PHJ	3^+^	3^+^	0	0


Jiangsu	Yancheng^a^	PCT	3^+^	3^+^	0	0


		PCH	3^+^	3	0	0


		PHK	3	3	0	0


		PHH	3	3	0	0


		FHH	3^+^	3^+^	0	0


Anhui	Chuzhou^a^	NCD	3	3	0	0


		PHT	3	3	0	0


		PHJ	3	3	0	0


	Suzhou^a^	PCH	3	3^+^	0	0


		PCQ	3^+^	3^+^	0	0


		PHK	3	3	0	0


		PHT	3	3+	0	0


		PHS	3	3	0	0


		NHK	3	3	0	0


Hubei	Xiangyang^a^	FCR	3^+^	3	0	0
		PCQ	3^+^	3^+^	0	0
	Xuelong^d^	PCR	3^+^	3	0	0
		PHR	3^+^	3	0	0
	Honghu^a^	DBQ	3^+^	3^+^	0	0
	Yanglingou^d^	DBG	3^+^	3^+^	0	0
	Jingzhou^a^	NBQ	3^+^	3^+^	0	0
	Gongan^b^	PCG	3^+^	3	0	0
		PCQ	3^+^	3	0	0
	Qianjiang^a^	PCH	3^+^	3	0	0
Shaanxi	Fufeng^b^	MCT	3	3	0	0
		TCG	3^+^	3^+^	0	0
Xinjiang	Mulei^b^	DBL	3^+^	3^+^	0	0
Yunnan	Yuxi^a^	PHS	3^+^	3^+^	0	0
		THJ	3	3	0	0
		MHK	3	3	0	0
		PCH	3^+^	3^+^	0	0
		PHK	3^+^	3^+^	0	0
		THK	3^+^	3^+^	0	0
		DCQ	3^+^	3^+^	0	0
		FCC	3	3^+^	0	0
	Midu^b^	PHJ	3^+^	3^+^	0	0
		MCH	3	3	0	0
		PHK	3^+^	3^+^	0	0
		PHS	3^+^	3^+^	0	0
		PHG	3^+^	3^+^	0	0
		MHJ	3^+^	3^+^	0	0
		PCG	3^+^	3^+^	0	0
		PCR	3^+^	3	0	0
		THT	3^+^	3	0	0
		PCC	3^+^	3^+^	0	0
	Lincang^a^	THT	3^+^	3^+^	0	0
		PCR	3	3	0	0
		FCG	3^+^	3	0	0
		PCH	3^+^	3^+^	0	0
Guizhou	Hezhang^b^	SHF	3	3^+^	0	0
		PHH	3^+^	3^+^	0	0
		PCQ	3^+^	3^+^	0	0
		FHT	3^+^	3	0	0
		SCG	3^+^	3^+^	0	0
		PHJ	3	3	0	0
		MCR	3	3	0	0
Sichuan	Zizhong^b^	FHK	3	3	0	0
		PHT	3^+^	3	0	0
		TBQ	3^+^	3^+^	0	0
	Yanting^b^	FBT	3	3	0	0
		FBS	3^+^	3^+^	0	0
		FHS	3^+^	3^+^	0	0
Chongqing (municipalities)	Tongnan^c^	PCR	3^+^	3	0	0
	Guagewan^d^	PCH	3	3	0	0
	Dashiban^d^	PBT	3^+^	3	0	0
Total: 13	40	50(102 samples)				

*^*a*^City; ^*b*^County; ^*c*^District; ^*d*^Village.‘+’ slightly larger uredinia than expected for the IT; ‘-’ slightly smaller uredinia than expected for the IT.*

### Identification of Leaf Rust in Nurseries

*Triticum aestivum*–*A. cristatum* 2P addition line II-9-3, the two BC_1_F_2_ backcross populations derived from *T. aestivum*–*A. cristatum* 2PL translocation lines 2PT-3(4DS.2PL), *T. aestivum*–*A. cristatum* 2PS translocation line 2PT-10(2PS.1AL) and Fukuho were planted in the nurseries in Xinxiang, Henan Province of China. The disease was assessed using the 0–4 infection type scoring system described by Roelfs (1992).

### Identification of Leaf Rust Resistance at the Seedling Stage

Two Chinese major dominant races THT and PHT (Liu and Chen, 2012), and other 48 races were used for inoculation. The *T. aestivum*–*A. cristatum* 2P addition lines, the translocation lines and the backcross populations were infected as described by Liu and Chen (2012). The races were inoculated onto 7-day-old seedling leaves of wheat plants planted in pots with low-nutrient soils. After inoculation, the plants were placed in a dew chamber at 18°C in the dark for 24 h. The inoculated wheat seedlings were then moved to a greenhouse at 24°C. The infection types (ITs) on the first leaves were recorded when Zhengzhou5389 was fully rusted, 15 days after inoculation. A 0 to 4 scale was used for ITs, as described by Roelfs (1992). Compared to the normal IT, larger and smaller uredinia were indicated with plus and minus signs. Plants with ITs 0-2 were considered resistant to leaf rust, whereas plants with ITs 3–4 were considered susceptible to leaf rust. Leaf rust infections were carried out at the greenhouse of the Institute of Plant Protection, Chinese Academy of Agricultural Sciences.

### Identification of Leaf Rust Resistance at the Adult Stage

The procedures for leaf rust resistance at the adult stage in the field were carried out as described by Qi et al. (2016). In this study, *T. aestivum* cv. ‘Zhengzhou5389’ was used as the susceptible control. One row of Zhengzhou5389 was planted every tenth row of tested materials to aid the spread of the spores within the trial (Qi et al., 2016). Zhengzhou5389 was also planted in the spreader rows perpendicular and adjacent to the test rows. The race THT was used for inoculation. Leaf rust infections were established by the spraying method at the wheat jointing stage. The plants were sprayed with an aqueous suspension of uredinia spores of *P. triticina* containing 0.05% Tween 20. Then, the inoculated plants were covered with plastic film, and the film was removed after 16 h (Song et al., 2016). ITs were recorded on the first leaf of each plant using a 0–4 scale when susceptible control Zhengzhou5389 were fully rusted. ITs were recorded in the same manner as the seedling stage.

### Molecular Marker Analysis

Genomic DNA was extracted using a modified CTAB method (Kidwell and Osborn, 1992; Li et al., 2017). A total of 82 *A. cristatum* chromosome 2P-specific STS markers which were developed based on the *A. cristatum* transcriptome sequences (Zhang et al., 2015, 2017) were mapped to 17 2P-specific bins, and the information of STS markers was described as Li et al. (2017). Of these, eight markers located on different 2P-specific bins were selected to detect the absence or presence of 2P chromosomal segments in the translocation lines and their backcross populations (**Table 4**). *A. cristatum* Z559 and II-9-3 were used as positive controls, whereas Fukuho and Zhengzhou5389 were used as negative controls.

**Table 4:** Primer sequences of the *Agropyron cristatum* 2P-specific STS markers for chromosome localization of the leaf rust resistance locus.

Marker name	Forward primer (5′–3′)	Reverse primer (5′–3′)	Tm (°C)	Chromosome bin
Agc52107	TCTTCCCCGACATCTCTCAC	CCGAAGGTAGTGGCGGTA	59	2PS (0.00–0.10)
Agc1846	ATGCATTTCTCCTGCCAGAC	GGACACTGGTGTTGATGTGC	59	2PS (0.50–0.95)
Agc17451	ATGATGTCGCCTGAATCTCC	ACACACCCCACAAAGAAAGC	59	2PL (0.47–0.52)
Agc32544	TTCGTCTTCGTCGGCAGACT	TGTCGCTGATCTCTCCAACG	59	2PL (0.52–0.60)
Agc4115	ACTCACGGTGCATGGTATGA	TTGTGCTGTGCGTGTGTAAA	59	2PL (0.66–0.86)
Agc3725	TGAGCAGAGACTTGGACTGG	TTCGTTGTGGCTTCAAAGTG	59	2PL (0.66–0.86)
Agc51058	TGGTCACATGGCAAGTTTACA	CGCCCTGATTTTTCATTCA	59	2PL (0.86–1.0)
Agc31475	GCTGGAGGAACTGTGATGGT	CTCAGGGTTCAAGTGCAACA	59	2PL (0.86–1.0)

*Tm, annealing temperature.*

The PCR amplification was performed as described by Li et al. (2017). The PCR amplification reactions were conducted in a total volume of 10 μl mixture containing 1.0 μl of template DNA (50 ng/μl), 1.0 μl of forward primer (2 μmol/l), 1.0 μl of reverse primer (2 μmol/l), 0.15 μl of 1 × *Taq* DNA polymerase (5 U/μl), 0.8 μl of MgCl_2_ (25 mmol/l), 0.8 μl of dNTP (2.5 mmol/l), 1.0 μl of 10 × buffer, and 4.25 μl of ddH_2_O. The cycling conditions were as follows: 94°C for 5 min followed by 38 cycles of 94°C for 30 s, 59°C for 30 s, and 72°C for 30 s, followed by a final 10-min extension at 72°C. The amplified products were separated by electrophoresis on a polyacrylamide gel with an acrylamide concentration of 8% s and visualized by silver staining.

## Results

### Leaf Rust Responses of the *T. aestivum*–*A. cristatum* 2P Addition Line and Translocation Lines in the Field

**FIGURE 2:**
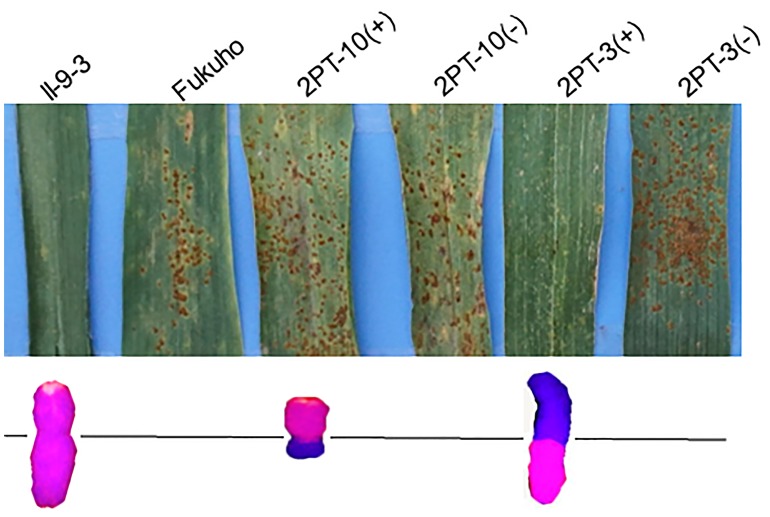
Disease responses to *P. triticina* of the *Triticum aestivum*–*Agropyron cristatum* 2P addition line and translocation line populations of wheat in the field. ‘+’ indicated the progenies of the translocation lines and their parents containing *A. cristatum* 2P chromatin; ‘–’ indicated the progenies of the translocation lines and their parents that do not contain *A. cristatum* 2P chromatin.

**FIGURE 3:**
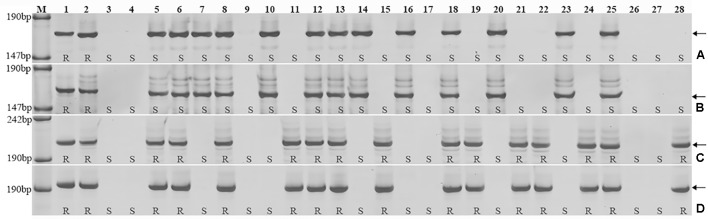
Amplification patterns of the *A. cristatum* 2P translocation lines using 4 STS markers specific for the *A. cristatum* 2P chromosome. *Agc1846* **(A)**, *Agc52107* **(B)**, *Agc3725* **(C)**, *Agc4115* **(D)**. *M*, pUC19 DNA/MspI (HpaII); *1 A. cristatum* accession Z559; *2* II-9-3; *3* Fukuho; *4* Zhengzhou5389; *5–28* the isolates of different populations. **(A,B)** Shows positive and negative plants of the isolates of 2PT-10, **(C,D)** shows positive and negative plants of the isolates of 2PT-3. The *arrows* indicate the diagnostic bands. R indicates resistance; S indicates susceptible.

*Triticum aestivum*–*A. cristatum* 2P addition line II-9-3, the two backcross populations derived from the cross between 2PL translocation lines 2PT-3, 2PS translocation line 2PT-10 and Fukuho were planted in the nurseries in Xinxiang during 2014 and 2015. Fukuho was highly susceptible to leaf rust, whereas II-9-3 was highly resistant (**Figure 2**). According to the leaf rust resistance results and molecular marker analysis (**Figure 3** and **Table 1**), in the 2PT-10 BC_1_F_2_ populations, all progenies were susceptible to leaf rust, regardless of the presence or absence of 2P-specific STS markers, suggesting that the leaf rust resistance locus is not located on the 2PS arm. In the 2PT-3 BC_1_F_2_ populations, all the progenies with 2P-specific STS markers were highly resistant to leaf rust, while other progenies of 2PT-3 without 2P-specific STS markers were as highly susceptible to leaf rust as Fukuho. The 2PT-3 translocation lines contained the whole 2PL arm, suggesting that the leaf rust resistance locus is located on the 2PL arm.

### Evaluation of the Resistance Spectrum and Availability of the 2PL Arm Against Leaf Rust

**FIGURE 4:**
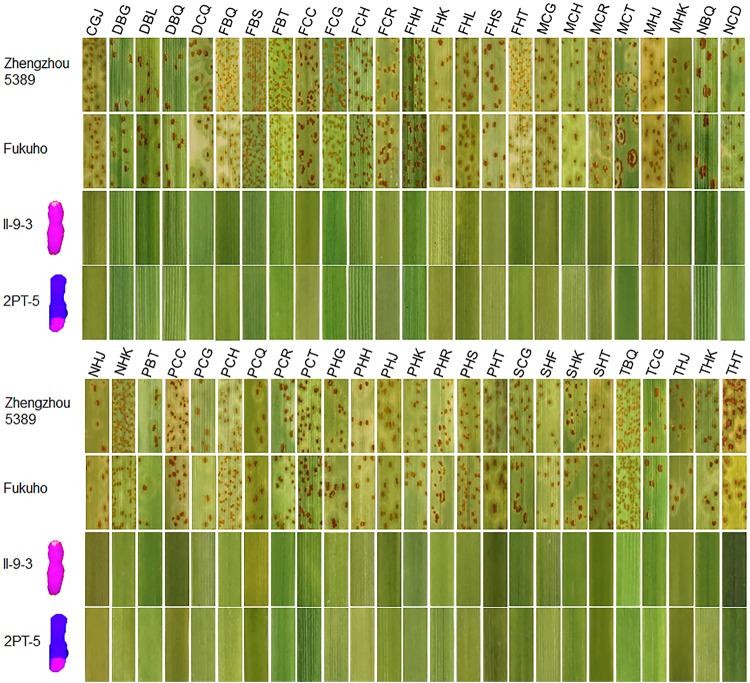
Disease responses to *P. triticina* of *T. aestivum*–*A. cristatum* 2P addition line II-9-3 and *T. aestivum*–*A. cristatum* 2P translocation line 2PT-5.

During 2015 and 2016, the responses of the translocation lines 2PT-5(3BS.L-2PL(0.6-1)), 2PT-3(4DS.2PL), and *T. aestivum*–*A. cristatum* 2P addition line II-9-3 were 0;, and all plants were characterized as nearly immune to leaf rust. To evaluate the resistance spectrum of *T. aestivum*–*A. cristatum* 2P II-9-3 and 2PT-5, 102 leaf rust samples (**Table 3**), which collected from 11 provinces, 1 autonomous region and 1 municipality of China in 2016 were used to inoculate II-9-3, 2PT-5, Fukuho and Zhengzhou5389 at the seedling stage in the greenhouse. Fifty races were identified from 102 samples by the State Key Laboratory for Biology of Plant Diseases and Insect Pests. Among these races, PHK, PHT, PCH, PHJ, PHS, THT, and PCG were found in different provinces. II-9-3 and 2PT-5 were nearly immune (IT = 0) to all 50 races, whereas Fukuho and Zhengzhou5389 were highly susceptible (IT = 3^+^ ∼ 4) to all 50 races at the seedling stage (**Figure 4** and **Table 3**). The disease responses to duplicate races from different locations showed the same ITs. These results suggested that II-9-3 and 2PT-5 have broad-spectrum leaf rust resistance and may be useful for wheat disease resistance improvement.

### Chromosomal Localization of Leaf Rust Resistance Locus on *A. cristatum* Chromosome 2P

**FIGURE 5:**
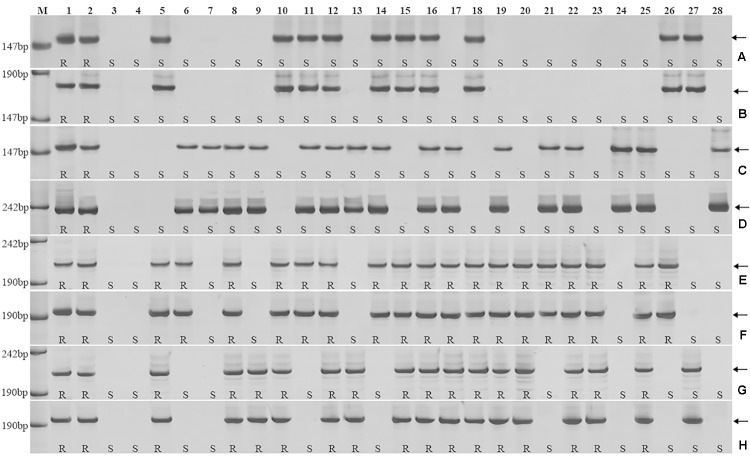
Amplification patterns of the *A. cristatum* 2PL translocation lines using 6 STS markers specific for the *A. cristatum* 2P chromosome. *Agc17451* **(A)**, *Agc32544* **(B)**, *Agc31475* **(C)**, *Agc51085* **(D)**, *Agc3725* **(E,G)**, *Agc4115* **(F,H)**. *M*, pUC19 DNA/MspI (HpaII); *1 A. cristatum* accession Z559; *2* II-9-3; *3* Fukuho; *4* Zhengzhou5389; *5–28* the isolates of different populations. **(A,B)** Shows positive and negative plants of the isolates of 2PT-6, **(C,D)** shows positive and negative plants of the isolates of 2PT-8, **(E,F)** shows positive and negative plants of the isolates of 2PT-5, **(G,H)** shows positive and negative plants of the isolates of 2PT-3. The *arrows* indicate the diagnostic bands. R indicates resistance; S indicates susceptible.

**FIGURE 6:**
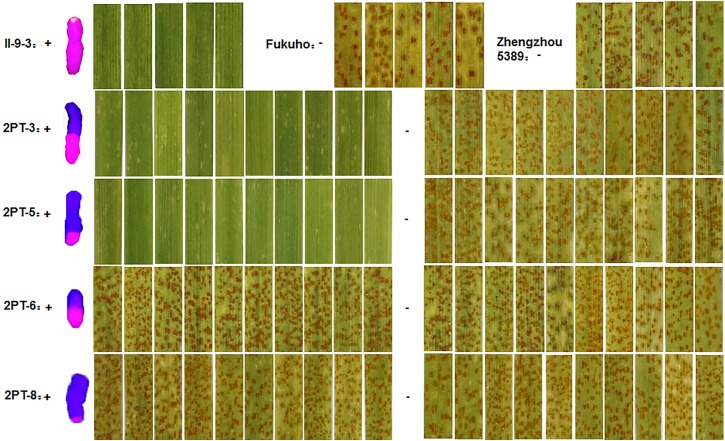
Disease responses to the *P. triticina* of the BC_2_F_2_ and BC_3_F_2_ populations of 4 *T. aestivum*–*A. cristatum* 2PL translocation lines and their parents at the seedling stage. ‘+’ indicates the progenies of the translocation lines and their parents containing *A. cristatum* 2P chromatin; ‘–’ indicates the progenies of the translocation lines and their parents that do not contain *A. cristatum* 2P chromatin.

**FIGURE 7:**
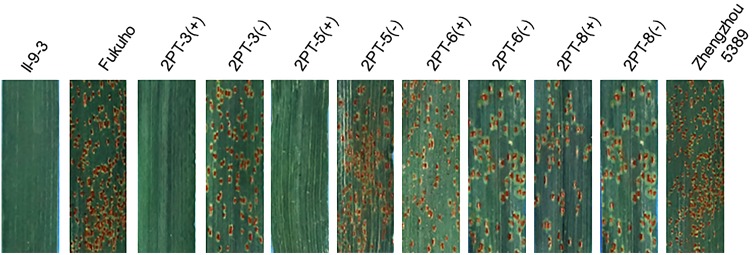
Disease responses to the *P. triticina* of the BC_2_F_2_ and BC_3_F_2_ populations of 4 *T. aestivum*–*A. cristatum* 2PL translocation lines and their parents at the adult plant stage. ‘+’ indicates the progenies of the translocation lines and their parents containing *A. cristatum* 2P chromatin; ‘–’ indicates the progenies of the translocation lines and their parents that do not contain *A. cristatum* 2P chromatin.

**FIGURE 8:**
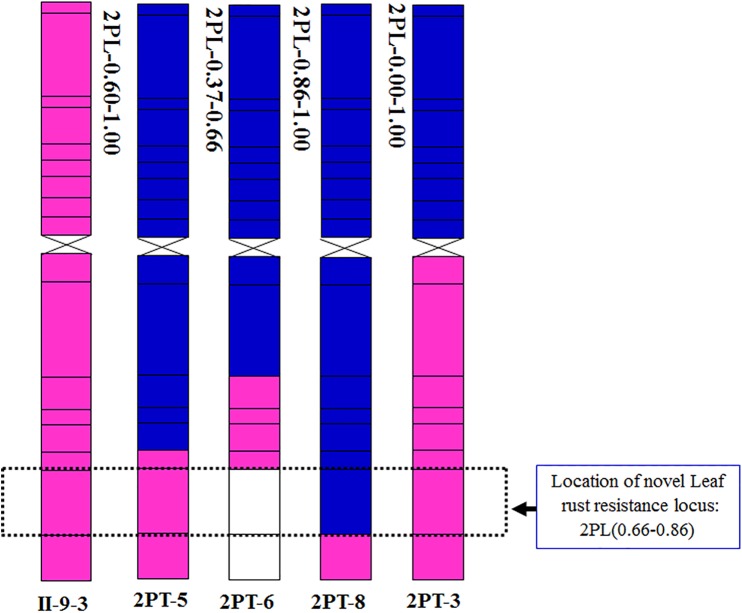
Chromosomal localization of the novel leaf rust resistance locus from *A. cristatum* chromosome 2P.

To further pinpoint the chromosomal segment containing the leaf rust resistance locus, three BC_2_F_2_ populations (2PT-5, 2PT-6, 2PT-8) and one BC_3_F_2_ population (2PT-3) derived from four translocation lines with different chromosomal segments of 2PL were constructed (**Table 2**). The major dominant races THT (Liu and Chen, 2012) was used to evaluate the leaf rust resistance of *T. aestivum*–*A. cristatum* 2P addition line II-9-3, Fukuho and the populations of 2PT-3, 2PT-5, 2PT-6, and 2PT-8 in the greenhouse and field, with Zhengzhou5389 used as the control. At the same time, the plants in each population were identified with the *A. cristatum* 2P-specific markers. According to the results of the molecular marker analysis (**Figure 5**) and the leaf rust responses of the four populations at the seedling stage (**Figure 6** and **Table 2**), the responses of *T. aestivum*–*A. cristatum* 2P addition line II-9-3 was 0, and all plants were nearly immune to leaf rust. The responses of Fukuho was 4, and all plants were as highly susceptible as the susceptible control Zhengzhou5389. In the 2PT-3 BC_3_F_2_ population, 142 positive plants were nearly immune (IT = 0) and 58 negative plants were highly susceptible (IT = 3^+^). In the 2PT-5 BC_2_F_2_ population, 145 positive plants were nearly immune (IT = 0) and 55 negative plants were highly susceptible (IT = 3^+^). In the 2PT-6 BC_2_F_2_ population, all of the 108 positive plants and 92 negative plants were highly susceptible (IT = 3^+^). In the 2PT-8 BC_2_F_2_ population, 110 positive plants and 86 negative plants were also highly susceptible (IT = 3^+^). These results suggest that the novel leaf rust resistance locus of the *T. aestivum*–*A. cristatum* 2P translocation lines is located in the chromosomal bin FL 0.66–0.86 of 2PL. The four populations of translocation lines were assessed for leaf rust resistance at the adult plant stage in the field. The results for the disease response to the *P. triticina* at the adult stage (**Figure 7** and **Table 2**), indicated that II-9-3 was nearly immune, whereas Fukuho was as highly susceptible as Zhengzhou5389 to leaf rust. In the 2PT-3 BC_3_F_2_ population, 106 positive plants of the isolates of 2PT-3 were nearly immune (IT = 0) and 53 negative plants were highly susceptible (IT = 3^+^). In the 2PT-5 BC_2_F_2_ population, 103 positive plants were nearly immune (IT = 0) and 50 negative plants were highly susceptible (IT = 3^+^). In the 2PT-6 BC_2_F_2_ population, all of the 64 positive plants and 62 negative plants were highly susceptible (IT = 3^+^). In the 2PT-8 BC_2_F_2_ population, 51 positive plants and 58 negative plants were also highly susceptible (IT = 3^+^). According to the evaluation of the leaf rust resistance of the BC_2_F_2_ and BC_3_F_2_ populations of the four *T. aestivum*–*A. cristatum* 2P translocation lines and their parents, II-9-3 and 2PT-5 were nearly immune to leaf rust at both the seedling and adult plant stages. Based on these results, the novel leaf rust resistance locus of *T. aestivum*–*A. cristatum* chromosome 2P is located in the chromosomal bin FL 0.66–0.86 of 2PL (**Figure 8**).

The leaf rust resistance locus is located in the same region as the powdery mildew resistance locus reported by Li et al. (2017). It is speculated that chromosomal bin FL 0.66–0.86 of 2PL carries two types of resistance loci: the leaf rust resistance locus and powdery mildew resistance locus.

### Evaluation of the Resistance Locus in the Chromosomal Bin FL 0.66–0.86 of 2PL

Recently, 2PT-3 was induced by ^60^Co-γ ray, leading to the creation of thirty-seven 2PL translocation lines with different chromosomal fragments and breakpoint positions. According to the results of molecular marker analysis, leaf rust and powdery mildew responses, eleven 2PL translocation lines displays nearly immune to leaf rust and high susceptible to powdery mildew; ten 2PL translocation lines displays high susceptible to leaf rust and high resistance to powdery mildew. Based on these result, different locus are responsible for resistance to powdery mildew and leaf rust.

## Discussion

Wheat leaf rust generally occurs in the world’s wheat-producing areas and is a major diseases in wheat affecting wheat production. Serious disease can significantly reduce yields. Recent increases in temperature and precipitation have gradually increased the prevalence of wheat leaf rust in the main wheat-production areas in China, especially in southwestern and northwestern China, the middle and lower regions of the Yangtze River, and the southern parts of the Huang-Huai-Hai River regions (Huerta-Espino et al., 2011). Yield losses were recorded in the Henan, Sichuan, Anhui, Gansu, and Shaanxi provinces in China in the year 2012 (Zhou et al., 2013). Liu and Chen (2012) studied the race and virulence frequency of *P. triticina* in China, and found that THT, PHT, THT, PHJ, THJ, and PCG were the main races in the most provinces and wheat areas. They also found that only *Lr9*, *Lr19*, *Lr24*, *Lr25*, *Lr28,* and *Lr29* were effective leaf rust resistance genes among the identified genes in China (Liu and Chen, 2012). Kolmer (2015) studied the leaf rust resistance genes and virulence of *P. triticina* from Sichuan, Hebei, Henan, Shandong, Anhui, Hubei, and Shanxi provinces of China. A total of 48 virulence phenotypes were detected and included FCBQQ, PCGLN, and PCGLL. *Lr1*, *Lr2c*, *Lr3*, *Lr26*, *LrB*, *Lr10*, *Lr11*, *Lr3bg*, *Lr20,* and *Lr14b* showed low disease resistance, indicating that the utilization value of these resistance genes was not significant. Most of the identified leaf rust resistance genes are race-specific resistance genes which easily to lose resistance in breeding (Kolmer, 2015). Because the disease continuously evolves and forms novel virulent races (Kolmer, 2005; Bolton et al., 2008), new varieties with different desirable genes are constantly needed to replace the varieties that have lost effectiveness (Herrera-Foessel et al., 2012). Therefore, mining novel resistance genes, breeding resistant cultivars by the utilization of leaf rust resistance genes, and using wheat cultivars with different resistance genes can extend the effective life of these genes and are of great significance to wheat breeding and production.

In this study, 50 races were collected from 11 provinces, 1 autonomous region and 1 municipality covering the main wheat-producing areas of the Huang-Huai-Hai River regions, the middle and lower regions of the Yangtze River, the northern and southwest parts of China. These races were used to inoculate plants to identify resistance locus. The novel leaf rust resistance locus were first confirmed in the *T. aestivum*–*A. cristatum* 2P addition line II-9-3 and 2PL translocation line 2PT-5 with broad-spectrum and strong availability. Lines of Thatcher wheat differing in the single leaf rust resistance genes *Lr1*, *Lr2c*, *Lr3*, *Lr16*, *Lr26*, *Lr3ka*, *Lr11*, *Lr17,* and *Lr30* were resistant to19, 7, 12, 28, 5, 26, 25, 10, and 25 of the 50 tested races, respectively. Therefore, the novel leaf rust resistance locus exhibited a broader spectrum than the nine genes above. *T. aestivum*–*A. cristatum* chromosome 6P addition line 4844-12 was highly resistance to leaf rust at the adult plant stage and the resistance locus was finally located on chromosome arm 6PS (Song et al., 2016). However, in the 6PS telosomic populations, the plants carrying *A. cristatum* chromosome 6P were not all resistance to leaf rust, in contrast to the resistance locus exploited in chromosome 2P in this study and this characteristic would affects the application of the gene from 6P in breeding. In conclusion, the novel leaf rust resistance locus have high utilization value and not only confer broad-spectrum resistance but also stronger and more stable than the resistance carried by *A. cristatum* chromosome 6P.

Many leaf rust resistance genes were found to have genetic linkages to genes conferring resistance to powdery mildew, stripe rust caused by *P. striiformis* f. sp. *tritici* (*Pst*) or stem rust caused by *P. graminis* Pers. f.sp. *tritici.* (*Pgt*) in wheat, such as *Lr19/Sr25*, *Lr24/Sr24/Yr71* (McIntosh et al., 1977), *Lr27/Sr2* (Mago et al., 2011), *Lr34/Yr18/Pm38/Sr57* (Krattinger et al., 2009), *Lr37/Yr17/Sr38* (Seah et al., 2001), *Lr46/Yr29/Sr58/Pm39* (Rosewarne et al., 2006; Lillemo et al., 2008), *Lr57/Yr40* (Kuraparthy et al., 2007), *Lr62/Yr42* (Marais et al., 2009), and *Lr67/Yr46/Sr55/Pm46* (Herrera-Foessel et al., 2014). The investigation and identification of leaf rust-resistance genes will allow these previously identified resistance genes to be used more effectively. *Lr34/Yr18/Pm38/Sr57* is a single wheat gene that confers durable and partial adult plant resistance against leaf rust, stripe rust, powdery mildew, and stem rust (Krattinger et al., 2009). This gene has been widely used in wheat breeding for more than one hundred years, and no pathogen adaptation has been observed so far, indicating that wheat with durable resistance genes in wheat can effectively prolong the effectiveness of resistance genes for different diseases (Krattinger et al., 2009; Keller et al., 2015). In this study, leaf rust resistance locus was located in the chromosomal bin FL 0.66–0.86 of 2PL using translocation line backcross populations that also carried powdery mildew resistance locus (Li et al., 2017). The leaf rust resistance locus, with broad-spectrum resistance, was high resistance to powdery mildew and nearly immune to leaf rust at both the seedling and adult stages. Chromosomal bin FL 0.66–0.86 of 2PL is probably an excellent gene segment with various disease resistance locus that are closely linked. *T. aestivum*–*A. cristatum* chromosome 2P translocation line 2PT-5, which is resistant to leaf rust and powdery mildew, is of great value for wheat resistance breeding.

*Triticum aestivum*–*A. cristatum* chromosome 2P translocation line 2PT-5 was not only showed nearly immune to wheat leaf rust and highly resistant to powdery mildew but also had many other desirable traits, such as a small and erect flag leaf, compact plant type, closely arranged spikelets, and long uppermost internode. *T. aestivum*–*A. cristatum* chromosome 2P translocation line 2PT-5 can be used in wheat breeding (submitted). Therefore, translocation line 2PT-5 is potentially useful for broadening the genetic basis of leaf rust resistance and provides valuable genetic material for wheat breeding. The combination of irradiation, backcrossing and other methods with the *ph1b* mutant will further delimit the chromosome 2P 0.66–0.86 segment smaller for the next step and even enable deeper study of leaf rust resistance gene(s) and powdery mildew gene(s).

## Author Contributions

WL conceived the research. BJ and TL performed the research. BJ wrote the paper. WL and HL created the translocation lines. LL created the *T. aestivum*–*A. cristatum* 2P addition line. HH, LL, JZ, XY, SZ, and XL participated in the preparation of the reagents and materials used in this study. All authors read and approved the manuscript.

## Conflict of Interest Statement

The authors declare that the research was conducted in the absence of any commercial or financial relationships that could be construed as a potential conflict of interest.
